# Severe Symptomatic Hypochloremia Associated with Rare Signet Ring Cell Carcinoma of the Ampulla of Vater: A Case Report

**DOI:** 10.7759/cureus.19492

**Published:** 2021-11-12

**Authors:** Atef Akoum, Rached Radwan, Said El Hage, Jad El Masri, Salah Ezzeddine

**Affiliations:** 1 Medicine, Lebanese University, Faculty of Medical Sciences, Hadath, LBN; 2 Internal Medicine, Lebanese University, Faculty of Medical Sciences, Hadath, LBN; 3 General Medicine, Lebanese University, Faculty of Medical Sciences, Hadath, LBN; 4 Neurosciences, Neuroscience Research Center, Lebanese University, Faculty of Medicine, Hadath, LBN; 5 Gastroenterology, Rafic Hariri University Hospital, Beirut, LBN

**Keywords:** ampulla of vater, electrolyte disturbances, signet ring cell type carcinoma, vomitting, hypochloremia

## Abstract

Hypochloremia is an electrolyte disturbance characterized by low serum concentration of chloride ions, often occurring in acute illnesses and characterized by nonspecific signs and symptoms. It rarely results from decreased intake and is predominantly due to either renal or extra-renal losses. We report a case of severely worsening symptomatic hypochloremia resulting from an extra-renal loss of chloride ion in a 58-year-old female patient presenting for prolonged protracted vomiting. Chloremia reached a surprising level of 48 mEq/L, the lowest level reported in the literature. The patient was eventually diagnosed with a rare signet ring cell carcinoma that occurred in the ampulla of Vater, leading to a malignant gastric outlet obstruction and causing extra-renal loss of chloride.

## Introduction

Chloride (Cl) is an inorganic halogen charged negatively, thus, considered an anion. It is distributed exclusively within the extracellular fluid compartment (ECF) and is closely related to sodium. In healthy individuals, chloride concentrations in the blood range from 96 to 106 mEq/L [1]. Though the second most abundant electrolyte in the serum after sodium and having a fundamental role in acid-base status, chloride is less emphasized in literature and under-evaluated and is not usually taught in distinct exclusive chapters in textbooks [2].

Hypochloremia, defined as a chloride level less than 95 mEq/L, is caused by renal or extra-renal problems. Among renal causes, two causes are considered the most common: loop diuretic and osmotic diuresis. The prominent extra-renal etiologies of hypochloremia are inadequate oral intake, loss of the ion via vomiting, nasogastric suction, diarrhea, and skin disruption (burn), and excess water gain as in congestive heart failure. Diagnosis will depend on urine chloride and sodium levels, which are elevated in hypochloremia due to renal causes [1,3,4]. Hypochloremia does not present with specific signs and symptoms, but the clinical scenario depends on the cause (renal or extra-renal). Physical findings reflect the hypovolemic status and the contraction of the extracellular fluid (ECF) volume (hypotension, tachycardia, and orthostatic hypotension) [1]. The physical findings in individuals with hypochloremia resulting from renal losses of sodium and chloride will be similar to those with extra-renal chloride losses. In these individuals, urine chloride and sodium levels will be raised, indicating renal losses of chloride (and sodium) despite evidence of ECF volume contraction [1].

We are presenting a case of a rare type of duodenal tumor causing malignant gastric outlet obstruction and subsequent severe vomiting leading to severe hypochloremia.

## Case presentation

A 58-year-old non-alcoholic female with a long-standing history of tobacco intake (28 pack-year) and no known comorbidities presented for increasing abdominal pain with frequent vomiting. History is significant for nausea and intermittent, non-projectile, non-bilious, post-prandial vomiting over the last four months, associated with fatigue, decreased oral intake, and considerable weight loss (approximately 20 kilograms).

Physical examination revealed an ill-looking cachectic female with regular heart sounds, poor bilateral air entry, and diffusely tender scaphoid abdomen with a palpable mass in the epigastric area. The patient was conscious, cooperative, and oriented to person, space, and time. No palpable lymph nodes were reported. Pedal pulses were palpable bilaterally, and lower limb edema was absent. No skin or mucosal lesions were noticed. Vital signs were within normal limits upon admission. ECG showed normal sinus rhythm. A chest x-ray showed clear lungs and free pleural spaces. Laboratory findings reveal metabolic alkalosis, with hypochloremia (65.8 mmol/L) and elevated alkaline phosphatase (138 U/L), as shown in Table 1.

**Table 1:** Laboratory findings upon the first presentation to the emergency department

WBC (thousand/microL)	8.08
Lymphocyte count (/microL)	800 (10%)
Hemoglobin (g/dL)	16.4
Platelets (/microL)	388,000
Sodium (mmol/L)	131
Potassium (mmol/L)	3.4
Chloride (mmol/L)	65.8
Bicarbonate (mmol/L)	39
Blood urea nitrogen (BUN) (mg/dL)	31
Creatinine (mg/dL)	1.01
Lipase (U/L)	105
Amylase (mg/dL)	235
Alanine transaminase (U/L)	12
Aspartate aminotransferase (U/L)	25
Alkaline phosphatase (U/L)	138
Gamma-glutamyltransferase (U/L)	83
Bilirubin (mg/dL)	0.65
International Normalized Ratio (INR)	1.0
Partial Thromboplastin Time (seconds)	33
Albumin (g/dL)	3.1
pH	7.51
Bicarbonate on blood gas (mmol/L)	33.8
PCO2 (mmHg)	42
PO2 (mmHg)	65
Oxygen saturation	95%

**Figure 1:**
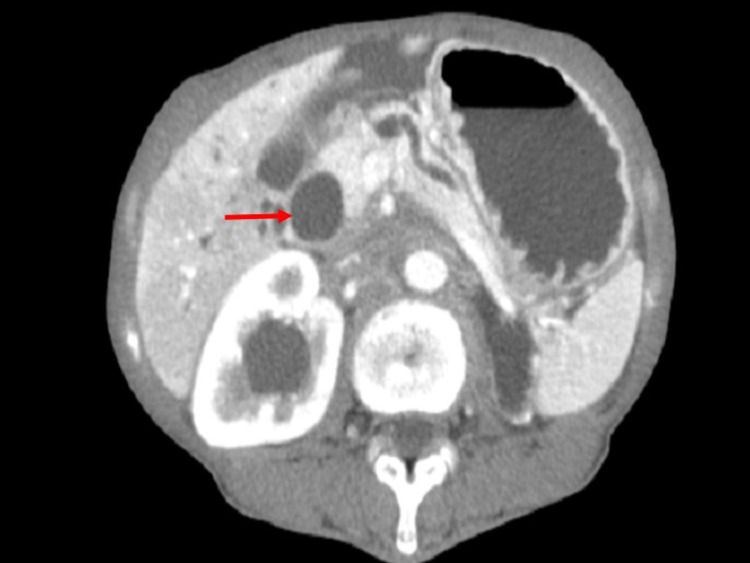
CT scan of the abdomen and pelvis showing distended common bile duct with dilation of the Wirsung duct

The patient received good intravenous (IV) hydration in the emergency department before admission to the hospital’s regular floor for further investigations. A peritoneal tap was performed and revealed a RBC value of 1210/microL, a pH of 8, a WBC count of 160/microL, a glucose level of 111 mg/dL, a lactate dehydrogenase level of 172 U/L, a protein level of 9.1 mg/dL, a negative culture, and an albumin level of 6.4 mg/dL. Imaging tests were subsequently ordered, including a CT scan of the abdomen and the pelvis with IV contrast (Figure 1).

**Figure 2:**
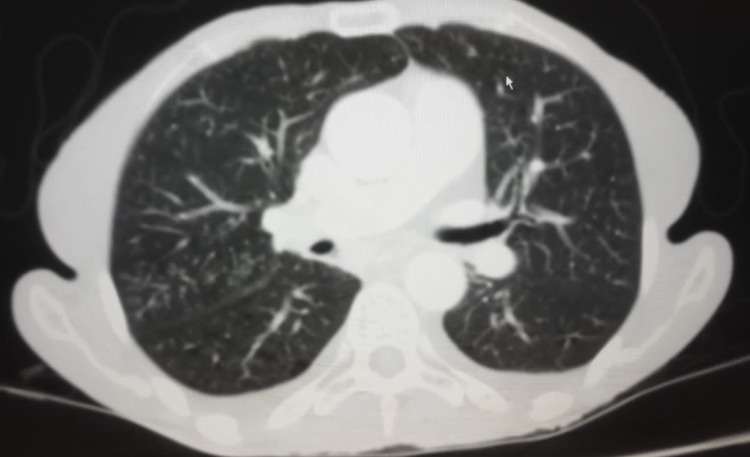
CT scan of the chest showing a tree in a bud appearance

Results showed a normal-sized liver with severe intra- and extrahepatic bile duct dilatation, associated with tortuous dilatation of the Wirsung duct. A diagnosis of an ampullary tumor was suspected. A thickening of the antrum of the stomach, submucosal edema, diffuse moderate to severe diffuse ascites, and peritoneal lining enhancement suggesting secondary peritoneal diseases were reported. The visualized bowels were collapsed. The right kidney showed mild pelvicalyceal dilatation with no calculi, while the left kidney was entirely atrophic. No lymphadenopathy was seen. Diffuse osteoporosis and degenerative modifications were reported in the L4-L5 disc space. A chest CT scan revealed diffuse centrilobular emphysematous changes with micro-nodular infiltrates, showing a tree in a bud appearance in the posterior segment of the right upper lobe and the posterior-apical part of the right lower lobe, as seen in Figure 2.

These findings were associated with mild bilateral pleural effusion and bibasilar atelectasis suggestive of infection/active tuberculosis and, less likely, micrometastasis. Paratracheal and subcarinal lymph nodes were also noted, and the largest node measured 1 cm on the short axis.

**Figure 3:**
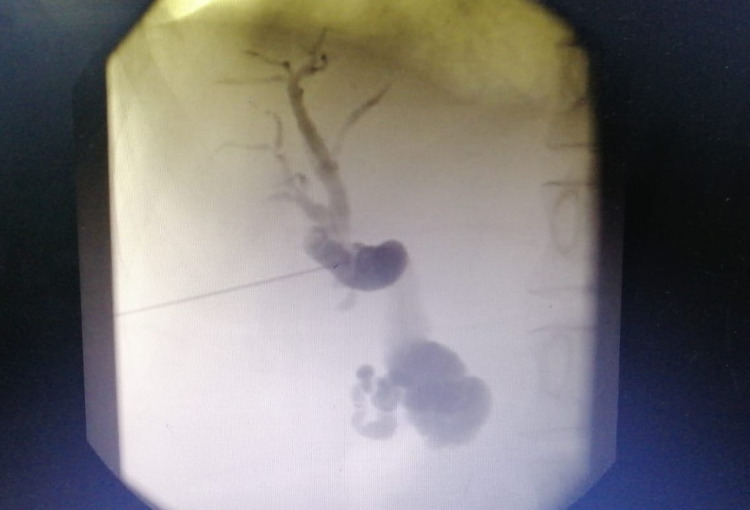
Endoscopic retrograde cholangiopancreatography (ERCP) showing obstructing mass

After the concerning CT chest results, bronchoscopy was done, and the resultant bronchoalveolar lavage (BAL) sample was negative for tuberculosis. Esophagogastroduodenoscopy (EGD) was scheduled on the second day of presentation (figure 3).

**Figure 4:**
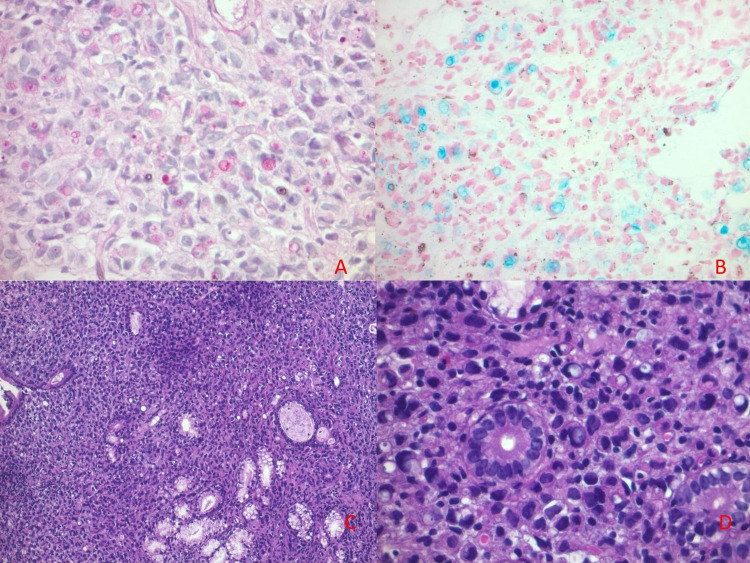
Histology of the obstructing mass, showing secretory vacuoles in the cytoplasm. A: Periodic acid-Schiff stain (PAS); B: Alcian blue stain; C: Low power hematoxylin and eosin stain (H&E stain), D: High power H&E stain

Gastric and duodenal biopsies were taken during the process (figure 4).

EGD findings included ulcerated esophagus with no signs of fungal infection, a negative periodic acid-Schiff (PAS) stain, and a chronic pangastritis associated with *Helicobacter pylori* with no intestinal metaplasia, glandular atrophy, dysplasia, or neoplasia. Furthermore, duodenal biopsy indicated a round cell poorly differentiated tumor, which was infiltrating the duodenal mucosa, morphologically compatible with signet-cell adenocarcinoma. Immunohistochemistry was recommended but not performed for financial purposes. Tumor markers revealed a normal-range alpha-fetoprotein level (1.9 ng/mL), a slightly elevated carcinoembryonic antigen (CEA) = 3.6 ng/mL and high levels of CA19-9 (70 U/mL). HIV, hepatitis B, and hepatitis C serologies were negative.

After the EGD and biopsy results, a gastrojejunostomy drain was inserted to relieve the gastric outlet obstruction, but the patient removed it, and she refused further medical interventions and was discharged against medical advice.

Three months later, the patient presents again to the emergency department with the same chief complaints but appeared more cachectic, toxic, and ill. She is complaining of generalized fatigue, lethargy, weakness, and vomiting. The patient had a decreased oral intake for the past five days due to persistent vomiting.

Upon presentation to the emergency department, the patient was hypotensive (blood pressure= 80/50 mmHg), other vital signs were within normal limits. Laboratory findings upon the second presentation are shown in Table 2 and show worsening hypochloremia (48.3 mEq/L), hypokalemia (2.2 mEq/L), hyperphosphatemia (7.79 mg/dL), a high BUN/creatinine ratio (42/1.5= 28) suggesting pre-renal injury. 

**Table 2:** Laboratory findings upon the second presentation to the emergency department

Calcium (mg/dL)	8.34
Magnesium (mg/dL)	2.12
Phosphorus (mg/dL)	7.79
Sodium (mEq/L)	127
Potassium (mEq/L)	2.2
Chloride (mEq/L)	48.3
Bicarbonate (mEq/L)	44.7
Blood urea nitrogen (mEq/L)	42
Creatinine (mg/dL)	1.5

Initial fluid recovery with normal saline was started. The patient eventually had a cardiopulmonary arrest, and the sinus rhythm was not restored during cardiopulmonary resuscitation. The patient had passed away.

## Discussion

Malignant gastric outlet obstruction occurs when gastric or pancreatic cancer causes a mechanical obstruction of the pylorus or the duodenum. The most common symptom is intractable vomiting and severe malnutrition. Nausea and vomiting are the cardinal symptoms of gastric outlet obstruction. In the early stages of occlusion, vomiting was intermittent. It usually occurs within one hour of a meal [4]. However, with the advancement of the disease, accompanied by the denial of treatment, the severity of the vomiting increased, leading to a hypochloremic state, which was predictable by other studies [5]. Palliative treatment options are based on surgical gastrojejunostomy and endoscopic enteral stenting [6]. In our case, as in other similar cases, severe recurrent vomiting caused this severe electrolyte disturbance (Chloride= 48 mEq/L). This chloride level is the lowest level reported in the literature.

Signet ring cell carcinoma (SRCC) is a malignancy that occurs in different organ systems such as the gastrointestinal tract, hepatic-pancreaticobiliary system, and urogenital system but primarily occurs in the stomach [7,8]. However, SRCC of the ampulla of Vater is extremely rare since only 39 cases are reported in the literature [9]. Although diagnosis is made by endoscopy and biopsy, a CT scan usually shows dilated common bile duct (CBD) without any mass, as seen in our case [10].

The only effective therapy is surgical removal because SRCC is less chemosensitive than non SRCC [11]. Furthermore, there is no specific chemo regimen for SRCC of the ampulla [12]. SRCC is usually associated with poor prognosis, and lymph node invasion is the most important prognostic factor [13]. Complete resection with negative margins and negative lymph node invasion is the most significant factor associated with improved survival in ampullary carcinoma [14]. 

It is well established that kidneys are responsible for the homeostasis of chloride ion, which is reabsorbed actively and passively throughout the tubules. Metabolic alkalosis is directly associated with hypochloremia as sodium bicarbonate reabsorption in the proximal convoluted tubule increases in hypovolemic settings with increased levels of angiotensin II [15]. This mechanism is seen in this patient since she had vomiting episodes and decreased oral intake, leading to severe hypovolemia and consequent contraction alkalosis.

Although a high chloride level in sweat test is used to diagnose cystic fibrosis [16], the literature suggests that hypochloremia is responsible for worse outcomes and higher all-cause mortality in patients with heart diseases admitted to the cardiac-care unit [17]. It is also considered a non-invasive predictor of mortality in patients suffering from pulmonary hypertension [18] and critically ill cirrhotic patients [19].

Recent studies confirmed the relation between hypochloremia and the severe outcome in a critically ill patient [20]. This level correlates well with higher acute physiology and chronic health evaluation II (APACHE II) scores and can indicate a poor prognosis [20]. Furthermore, an increased risk of one-year mortality is seen with chloride abnormalities (hypo and hyperchloremia) at discharge regardless of chloride level during admission [15].

Concerning the treatment of hypochloremia, hydration status should be assessed, and replacement of fluid loss with normal saline should be initiated promptly, in addition to nutritional assessment and adequate energy intake through the enteral route [20].

To the best of our knowledge, this combination of SRCC of the ampulla of Vater with this severely low chloride level constitutes the first reported case in the literature.

## Conclusions

Hypochloremia is an essential indicator of illness severity, although not widely used in practice. It indicates a worse prognosis in many diseases and is caused by renal or extra-renal losses. While vomiting is a chief cause of low chloride levels, it is rarely associated with malignant gastric outlet obstruction, such as in this patient diagnosed with SRCC in the ampulla of Vater. Electrolyte disturbances should be closely monitored and corrected in any gastric outlet obstruction, as it affects prognosis.
